# Exploring associations between active school environments and children’s physical activity, mental health and educational performance in Greater London primary schools: the Health and Activity of Pupils in the Primary Years (HAPPY) study protocol

**DOI:** 10.1136/bmjopen-2025-103463

**Published:** 2025-07-28

**Authors:** Bina Ram, Nancy Gullett, Amina Benkhelfa, Mark Cunningham, Mansour Taghavi Azar Sharabiani, Esther van Sluijs, Nadia Siddiqui, Melvyn Hillsdon, Carolyn Summerbell, Miranda Pallan, Sonia Saxena

**Affiliations:** 1 Department of Primary Care and Public Health, Imperial College London, London, UK; 2 MRC Epidemiology Unit, Cambridge University, Cambridge, UK; 3 Department of Education, Durham University, Durham, UK; 4 FUSE, the Centre for Translational Research in Public Health, Newcastle University, Newcastle upon Tyne, UK; 5 Department of Sport and Health Sciences, University of Exeter, Exeter, UK; 6 Department of Sport and Exercise Sciences, Durham University, Durham, UK; 7 Public Health, Epidemiology and Biostatistics, University of Birmingham, Birmingham, UK

**Keywords:** Schools, Community child health, Physical Fitness, PUBLIC HEALTH

## Abstract

**Abstract:**

**Introduction:**

School environments that encourage children to be physically active can embed lifelong positive health behaviours and contribute towards reducing health inequalities. The Health and Activity of Pupils in the Primary Years (HAPPY) study aims to: (1) explore the extent to which the WHO criteria for creating active school environments are implemented by primary schools and (2) examine associations between active school environments and children’s physical activity, mental health and educational performance.

**Methods and analysis:**

The HAPPY study is a quasi-experimental study comprising: (1) a survey of state-funded Greater London primary schools to identify implementation of the WHO’s six criteria and (2) a cross-sectional study to examine associations between schools’ active environment score (derived from the school survey) and pupils’ physical activity, mental health and educational performance. For our cross-sectional study, we will recruit up to 1000 year-three children (aged 7–8 years). Our primary outcome is accelerometer (GENEActiv) assessed physical activity, our secondary outcomes are parent-reported child mental health (Strengths and Difficulties Questionnaire) and teacher-reported educational performance (age-related expectations). Using multilevel mixed-effects regression models, we will examine associations between the active environment score and physical activity. Physical activity will be included as a measure of acceleration and also different intensities (light, moderate, vigorous). We will repeat this analysis to examine associations between the active environment score and mental health and educational performance. We will adjust for school characteristics and area-level deprivation and include pupil characteristics (eg, sex, ethnic group) as covariates. Clustering at the school level will be included as a random effect.

**Ethics and dissemination:**

Ethical approval has been obtained from Imperial College Research Ethics Committee (ref: 6800895). Findings will be disseminated through a summary report to all participating schools, peer-reviewed publications, presentations at national and international conferences and National Institute for Health and Care Research policy briefings.

**STRENGTHS AND LIMITATIONS OF THIS STUDY**

- This protocol describes an observational quasi-experimental study to understand schools’ policies and practices for creating active school environments.
- By targeting all schools in Greater London, the survey will reflect a diverse urban conurbation and multi-ethnic primary school population.
- The use of accelerometers in the cross-sectional study will provide accurate measures of physical activity and avoid potential bias of self-reporting.
- Multilevel mixed-effects regression models will include clustering of pupils by school, to allow for accurate standard errors and parameter estimates when testing for associations between active environment scores and outcomes.
- Low response rates to the school survey may affect generalisability of findings to other diverse urban conurbations.

## Introduction

 Regular physical activity during childhood is promoted by the WHO and governments worldwide to encourage lifelong health.^1 2^ Schools are among the most important settings for creating active environments for young people to help meet global targets to reduce physical inactivity by 15% by 2030.^2^,^4^ Schooling is compulsory for all children in many high-income settings and where most children spend around a third of their weekday waking time. Thus, whole-school approaches to address physical activity have the potential to level health inequalities^5^,^7^ and have been promoted in global health policies including the Global Action Plan on Physical Activity 2018–2030,^2^ Commission on Ending Childhood Obesity^8^ and UNESCO.^9 10^

UK guidelines recommend that children and young people (aged 5–18 years) should engage in at least 60 min of moderate-to-vigorous physical activity per day, of which 30 min should take place during the school day.^11^ Evidence from England suggests that 30% of children aged 5–16 years are physically active for less than 30 min per day.^12^ Furthermore, children aged 5–11 years, those from lower-income households and those from ethnic minority groups are the least physically active.^12^ Hence, a major implementation gap exists between policy recommendations and practice.

In 2021, the WHO published a policy briefing of promoting physical activity through a whole-school approach^13^ and a toolkit^3^ for schools and colleges to encourage children to be physically active. The development of this toolkit was informed by evidence-based interventions for increasing physical activity among children in school settings.^3^ The toolkit recommends six domains to encourage physical activity; these are: (1) quality physical education, (2) active travel, (3) opportunities to be physically active before and after school, (4) opportunities to be physically active at breaks and lunch, (5) active classrooms and (6) inclusive physical activity approaches for those with additional needs. These approaches need to ensure participation of most, if not all, children and the inclusion of those who may face additional barriers to being physically active outside school.^3 4^

The aim of our study is to explore the implementation of physical activity policies based on the WHO’s recommendation of a whole-school approach and to examine associations between a derived active environment score and children’s physical activity, mental health and educational performance.

Our aim is underpinned by the following research questions:

1. Which of the WHO’s policies and practices are currently implemented by primary schools in Greater London to create a physically active school environment?
2. Do schools with a higher active school environment score have better physical activity, mental health and educational performance among their pupils?
3. How equitable is the level of an active school environment across subgroups of children including those living in deprived areas, minority ethnic groups and those with disabilities?

## Methods and analysis

### Study design

**Figure 1:**
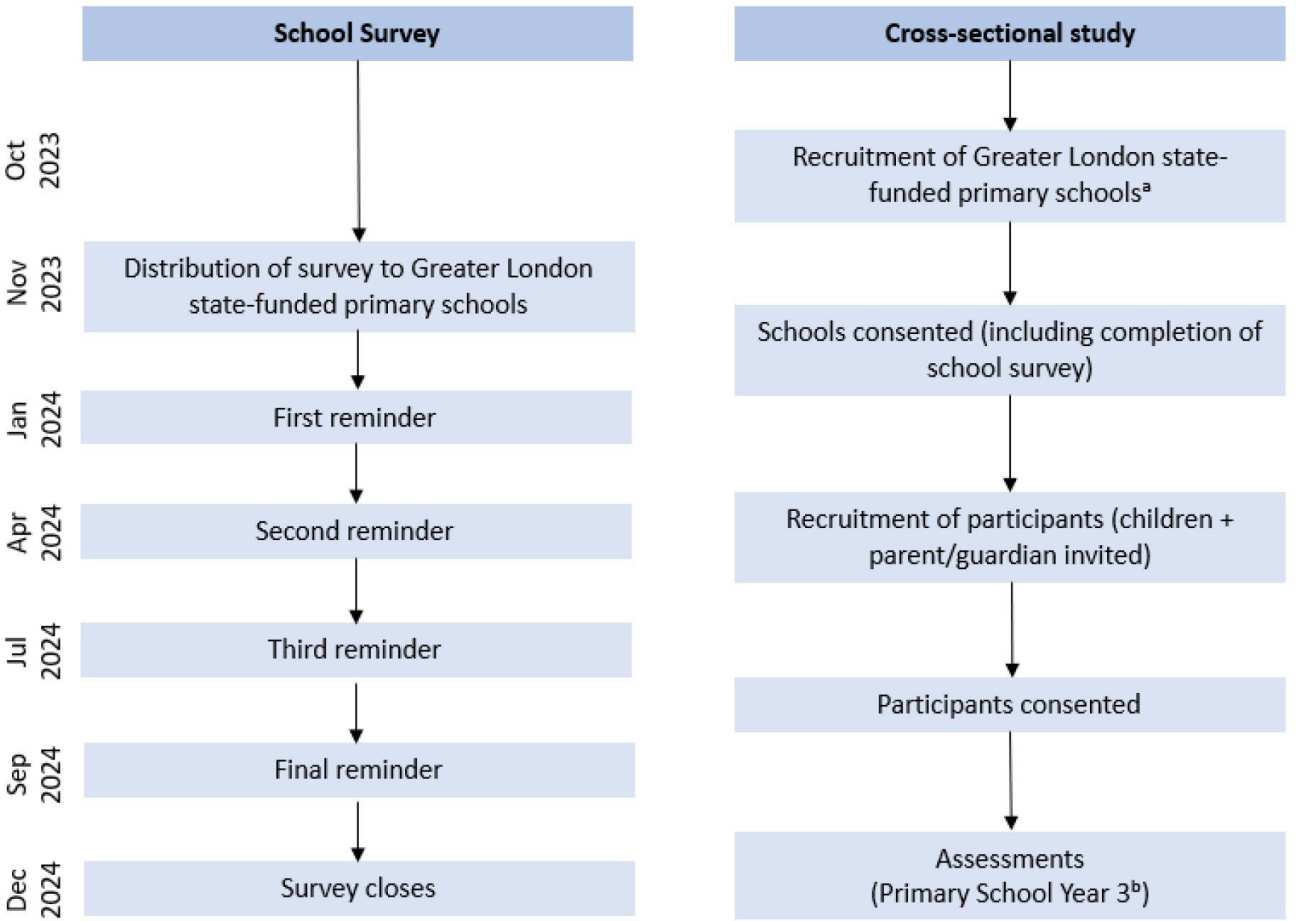
Health and Activity of Pupils in the Primary Years study design. ^a^Schools were recruited and children assessed throughout the data collection period; ^b^children aged 7–8 years.

The Health and Activity of Pupils in the Primary Years (HAPPY) study is an observational quasi-experimental study comprising two components involving primary data collection between October 2023 and December 2024 and was conducted simultaneously (figure 1). The data collection is completed and is being processed and cleaned in preparation for the analysis. The first component of the study was a survey of Greater London state-funded primary schools which assessed the implementation of physical activity policies and practices measured against the WHO recommendations. The second component was a cross-sectional study which measured children’s physical activity (using accelerometers), mental health (parent-reported) and educational performance (teacher-reported).

### Study setting and population

Our school survey was distributed to all state-funded primary schools in Greater London (n=1826). For our cross-sectional study, primary schools were recruited to participate, through which we invited children in year three (aged 7–8 years; one of the age groups identified as the least active in the primary school years^12^) and their parent/guardian to participate. All children in year three were eligible for participation, but participation was optional. Completion of both consent (adult) and assent (child) was required to participate in the study. Our target, based on our previous work,^14^ was to recruit up to 1000 children from approximately 40 schools across Greater London.

### Procedure

#### School survey

Details of all state-funded primary schools were derived from a schools database that we created previously^15^ and updated with details to include new schools. All school details are publicly available from the UK government website.^16^ We distributed paper versions of our survey by post (including a freepost envelope to return the completed survey) in November 2023 and sent four reminders by email (January 2024, April 2024 and July 2024) to encourage survey completion; a final reminder, by post, took place in September 2024 (see figure 1). The survey (both by post and email) was addressed for the attention of the head teacher, but any teacher best placed to answer the questions could complete the survey. The survey closed in December 2024.

#### Cross-sectional study

We worked with the National Institute for Health and Care Research (NIHR) Clinical Research Network and Schools Research Network who supported the recruitment of primary schools across Greater London. We targeted state-funded primary schools only. These schools are local authority maintained schools (academy converter, academy sponsor led, community, foundation, free schools, voluntary aided and voluntary controlled). State-funded primary schools receive funding through their local authority or directly from the government and they must follow a national curriculum (a set of subjects and standards for children to learn the same things).^17^ Schools were initially contacted by email and those providing an expression of interest were contacted by phone to discuss participation. On verbal agreement, schools were sent a consent form and information sheet (and the school survey if applicable). Through participating schools, we invited children and their parent/guardian to take part in the study by distributing a recruitment pack to the children. The recruitment pack contained a cover letter with information about the study (including details about the study team and contact details), a reply slip (for parents to indicate their participation and to provide their contact details), a parent/guardian information sheet, a children’s information sheet, two consent forms (a parent/guardian consent form and an adult consent form) and a child assent form. Obtaining parent/guardian contact details (address, phone number, email address) through the reply slip allowed the study team to contact the parent/guardian directly to follow-up any missing data. The information sheet explained what participation involved and that all data needed to be collected for the child to receive the monetary incentive (£5 voucher) as a thank you. The completed recruitment packs were returned to the school and collected by the study team 1 week after distribution. Those who had completed the reply slips and consent/assent forms were included in the cross-sectional study. All General Data Protection Regulations were followed. Recruitment of schools commenced in October 2024 with the first data collection taking place in January 2024. All data were collected by the end of December 2024.

### Consent

#### School survey

Completion of our school survey did not require consent; completion of the survey assumed schools were happy for the data they were providing to be used. No identifiable information about the school, except the borough which the school is in, will be reported. The school survey and cover letter explained how survey responses will be used.

#### Cross-sectional study

Prior to any data collection for our cross-sectional study, written and informed consent was obtained from schools and parents/guardians, and assent was obtained from children. Schools agreeing to participate were required to complete a consent form signed by the headteacher. At the convenience of the school, a study researcher visited the school to deliver a talk to year three children for 5–10 min about the study. The recruitment packs were given to the children to take home. The recruitment pack included consent and assent forms for parents/guardians and children, respectively. Those who returned completed consent forms (both parent/guardian and their child needed to provide consent and assent) were included for participation in the study. A child who provided assent but their parent/guardian did not was not eligible (and vice versa).

### Data collection: cross-sectional study

There was one time point of data collection per school. Children were visited by a study researcher at their school, who provided the children with an explanation of study involvement and what they needed to do (reiterating the information provided in the information sheet that was included in their recruitment packs) and measured the childrens height and bio-impedance. The children were fitted with the wrist-worn accelerometer on their non-dominant wrist and given a data-collection pack to take home that included instructions of how to wear the accelerometer, duration of wear (7 days a week and worn at all times—not to be taken off during the 7-day wear period), what to do if the accelerometer became uncomfortable to wear and how to contact the study team if the accelerometer was damaged or lost. The recruitment packs also included a paper version of the parent/guardian and child questionnaires (questionnaires were also available to complete online via a link/quick response (QR) code). The front of the pack included the date on which the child should take off the accelerometer and return the accelerometer and completed questionnaires back to the school. The class teachers of participating children were given a questionnaire (paper version) to complete about each participating child’s educational performance. Accelerometers and all questionnaires were collected from the school by the study researcher. Each child who returned their accelerometer and completed questionnaires received a certificate and monetary incentive (Amazon voucher). The study researcher followed up directly with the parent for any accelerometers that were not returned and/or incomplete questionnaires.

### Outcomes and measures

**Figure 2:**
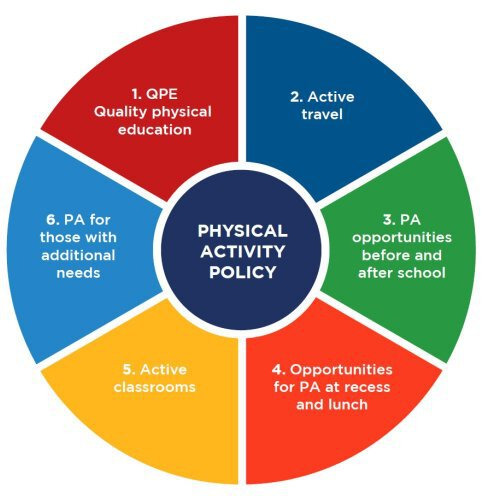
WHO’s six domains of a whole-school approach to promote physical activity (PA) through schools. Source: WHO, Promoting Physical Activity through Schools: a toolkit. QPE, quality physical education.

A full description of each WHO’s toolkit six key domains (figure 2)^3^ to promote physical activity during the school day is provided in online supplemental file 1.

#### School survey

Based on the WHO’s six domains of a whole-school physical activity approach (figure 2),^3^ we consulted with teachers to identify schools’ current practices and policies that support a physically active learning environment. We extracted relevant questions from previous studies of children’s physical activity, including SPEEDY,^18^ WAVES^19^ and GoActive,^20 21^ to capture whether schools support the delivery of a whole-school approach. We piloted the survey by asking primary school teachers to provide feedback on flow, response options, language, ease of understanding and the duration of completion. Any changes were reviewed and agreed by the authors and teachers. Two formats of our survey were created, an online version (using Qualtrics software) accessible via a link or QR code and a paper-based version (online supplemental file 2). Based on the responses from the survey, we will derive an ‘Active Environment Score’ for each school.

#### Cross-sectional study

We have collected data on accelerometer-measured physical activity, and through questionnaires, we have collected data on children’s mental health (reported by the parent/guardian) and educational performance (reported by class teachers), detailed in online supplemental file 3. Our outcomes were informed by a core outcome set for assessing school-based physical activity interventions which was jointly produced by the authors and multi-national stakeholders.^22^

#### Physical activity

We used the GENEActiv (ActivInsights Ltd, Kimbolton, Cambridgeshire, UK) wrist-worn accelerometers to measure objective physical activity which has been validated for use among children aged 5–8 years.^23 24^ The GENEActiv is a triaxial STMMicroelectronics accelerometer with a dynamic range of +/-8 g (1 g=9.81 m/s^2^) where *g* represents gravitational unit. It collects raw data on acceleration, is water-resistant (ie, can be worn 24/7) and has been reported to have higher compliance rates than hip-worn accelerometers.^25^ For the HAPPY study, acceleration was sampled at 85.70 Hz to allow for a measurement period of 9 days, capturing a full week (7 days) of data. Data will be extracted using the GENEActiv software and raw data will be processed using 5-second epochs using the GGIR R-package.^26^

#### Mental health

We used the Strengths and Difficulties Questionnaire (SDQ),^27^ a measure of children’s mental health. The SDQ is a brief behavioural screening questionnaire for children and young people aged 2–17 years. It has five subscales which each contain five items that assess emotional symptoms, conduct problems, hyperactivity, peer relationship problems and prosocial behaviour. It has been extensively validated^28^ and is one of the most widely and internationally used measures for assessing children’s mental health.^29^ Scores are derived for each of the subscales, higher scores indicating more difficulties. A ‘total difficulties’ score is calculated by summing the scores for four of the five subscales (excluding the prosocial behaviour subscale).

#### Educational performance

Teachers rated each participating child’s ability for reading, writing and mathematics on a 5-point Likert scale ranging from 1 (below expected levels) to 5 (above expected levels).^30^ Ratings are based on the UK’s National Curriculum’s ‘age-related expectations’ which are the standard expectations defined by threshold descriptors indicating the level pupils should be achieving by the end of primary school.^31^ Teachers also rated the child’s concentration and focus in class using a 5-point Likert scale where scores ranged from 0 (never has difficulties) to 4 (always has difficulties).

### Other measures

We measured anthropometry of all participating children. Height was measured to the last complete mm with a portable stadiometer (Seca, Leicester). A Tanita DC-240 MA Body Composition Analyser (Tanita; Tokyo, Japan) was used to measure weight to the nearest kg and leg-to-leg bioelectrical impedance from which fat-free mass and fat mass will be estimated. Body mass index (BMI) will be calculated as weight (kg)/height squared (m^2^), and fat mass percentage will be calculated as 100× (fat mass in kg/weight in kg).

### Public involvement and engagement

We consulted with primary school teachers to produce our school survey who were involved with the design of questions and approved the final version of the survey. Our questionnaires for the cross-sectional study aimed at parents/guardians and children were based on those used in a previous study which had been piloted.^14^ We are involving teachers, parents and children throughout the duration of our project to ensure that our findings will be translated to schools and relevant community groups.

We have consulted with and will continue to consult with our wider NIHR collaborative groups including our School for Public Health Research Member Collaborators, Advisory Group Members and Practice and Policy Collaborators to increase project impact and produce policy briefings.

### Sample size

The power calculation for this study used a multi-level model to account for the hierarchical structure of the data, with pupils (level 1) nested within schools (level 2). The model included random intercepts to capture variability between schools and examined the relationship between Active Environment Score (maximum score 50 points) and school day physical activity (in min). The analysis adjusted for key covariates such as sex (with females expected to engage in fewer than 10 min of physical activity), ethnicity, weekend physical activity, bioimpedance (used as a proxy for body composition), area deprivation and month of assessment (with higher activity expected during warmer months).

Based on the data collected to date, the sample size analysis included 600 pupils from 20 schools, with an average of 30 pupils recruited per school. The sample size of 600 pupils was the minimum number required for our power calculations; however, we aimed to recruit 1000 children to account for potential data losses (i.e., lost accelerometers, incomplete questionnaires, etc). 17 schools had preobserved Active Environment Scores ranging from 26 to 41, while the scores for the remaining three schools were simulated within this range for consistency.

**Figure 3:**
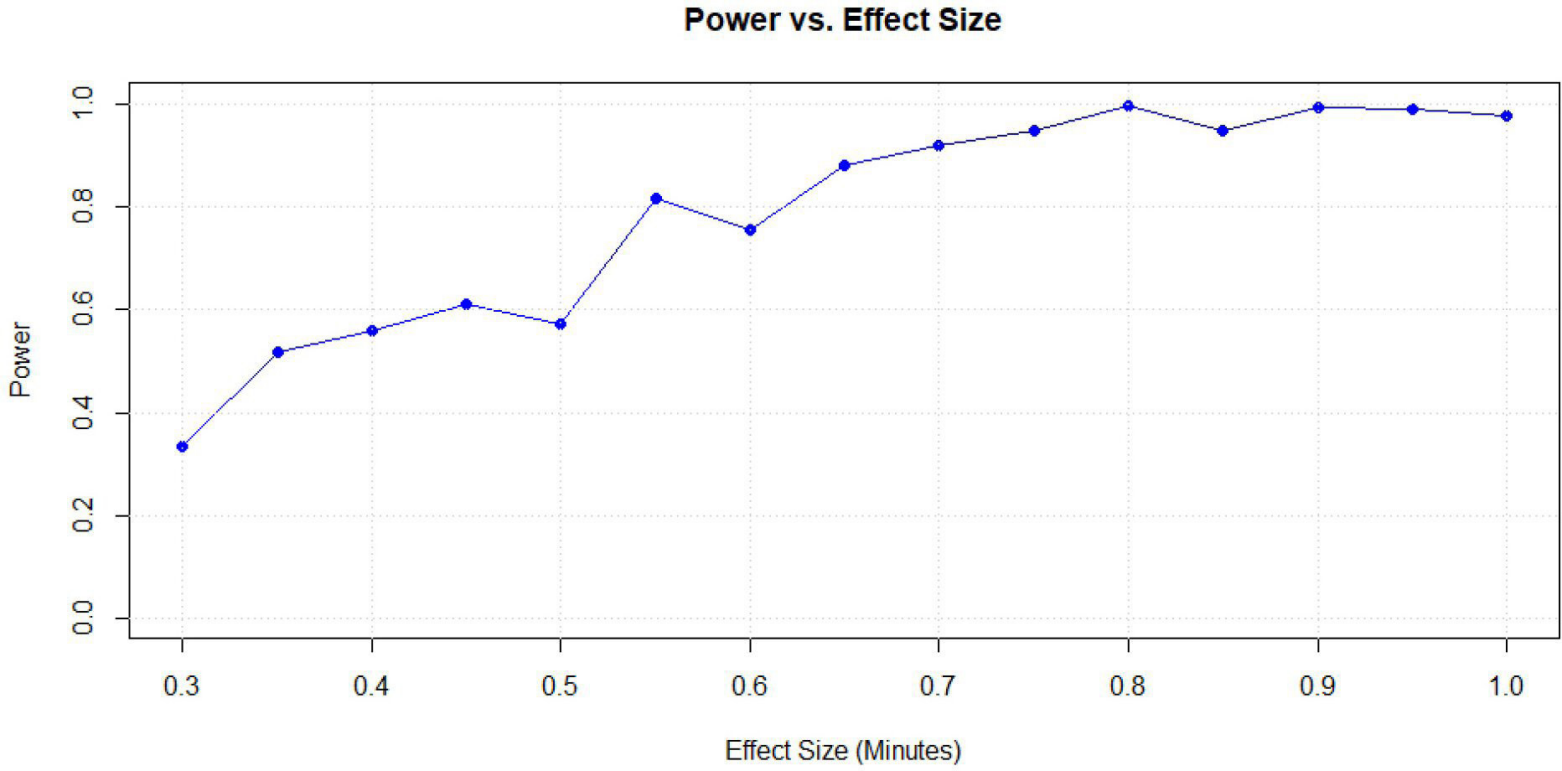
Power versus effect size for the Health and Activity of Pupils in the Primary Years study.

Power calculations were conducted using Monte Carlo^32^ simulations. We based our effect size on 60 min of physical activity per day (recommended global guidelines), of which 30 min should take place during the school day. We hypothesised that schools’ Active Environment Score would contribute to changes in the baseline; thus, an effect size of 0.50 would be clinically significant. However, we also explored a range of effect sizes, from 0.30 min to 1.00 min per unit increase in the Active Environment Score. The results indicated that the study achieves 81% power when a unit increase in the Active Environment Score corresponds to a 0.55-minute increase in physical activity. For increases of 0.70 min or more per unit of the Active Environment Score, the study achieves over 90% power, demonstrating a strong capability to detect these changes (see figure 3). These findings indicate that the study is well-powered to detect meaningful differences in physical activity when the Active Environment Score has a measurable impact, particularly for changes of 0.55 min or more per score unit. This provides confidence that the study design is adequate for identifying associations of this magnitude under the current assumptions.

The statistical power of the multi-level model, based on 600 pupils nested within 20 schools, is shown across a range of effect sizes (minutes of physical activity per unit increase in the Active Environment Score). While power generally increases with larger effect sizes, the minor irregularities observed in the power curve can be attributed to the stochasticity inherent in Monte Carlo simulations, where random variability in the data leads to slight fluctuations in the results.

### Statistical analysis

All data collection for the study has been completed. The data are being processed and cleaned in preparation for the analysis. We will characterise features of our study population including child sex, ethnicity and adiposity. We will report characteristics of schools including school size, ethnic mix, Ofsted rating, percentage eligible for free school meals and area-based deprivation. We will present school and pupil level baseline characteristics by active environment scores with summary measures (totals, percentages, means and SD, medians and IQRs as appropriate).

The main analysis will treat the main outcome (physical activity during the school day) as a continuous variable. Decisions on valid accelerometer data to include in the analysis will be made when reviewing the data. We will use mixed-effects regression models to estimate the association between physical activity acceleration and the active school environment score. We will include pupil-level covariates (sex, ethnicity) and account for clustering at the school level by including random effects. Covariates will be included in the model in stages to assess how much each contributes to diluting or increasing the effect. To measure equity, the primary analysis will adjust for pre-specified school characteristics including school size, pupils eligible for free school meals, pupils whose first language is not English, children with Special Educational Needs and Disabilities and area-level deprivation. Sensitivity analysis will include clustering at the class-level within the school.

Results will be reported with a 95% CI. Mixed effects regression models as described for physical activity will be repeated for the outcomes of mental health and educational performance. All analyses will be carried out using Stata v18.5 and R statistical software. We will report our findings in line with the Strengthening the Reporting of Observational Studies in Epidemiology^33^ and Logical Explanations & Visualizations of Estimates in Linear mixed models guidelines^34^ for reporting cross-sectional studies.

## Ethics and dissemination

Ethical approval has been received by Imperial College London Research Ethics Committee (reference: 6800895). All data collected have been pseudoanonymised (to allow for the removal of data for any participant withdrawing after data have been collected) and held at Imperial College London on secure servers and encrypted with access limited to the study team only. Ethical approval for this research study was granted on the basis that the data were stored securely and only accessible to the research team; therefore, the data will not be uploaded or shared via a repository. However, requests for access to deidentified data may be directed to the principal investigator (SS) who is responsible for data integrity and management.

The findings of this study will be disseminated through scientific peer-reviewed journals, national and international conference presentations, policy briefings and a summary of findings (eg, report, infographic) will be shared with the participating schools.

## Discussion

Schools play a critical role in helping embed the importance of PA by promoting active health behaviours, increasing opportunities for children to be physically active and creating active environments that encourage children to be physically active throughout the school day.^4 35 36^

The HAPPY study will be the most comprehensive exploration to date of how whole-school physical activity practices and policies are implemented in real-world settings according to the WHO key domains, whether promoting active environments is associated with children’s increased physical activity, improved mental health and educational performance and whether opportunities to be physically active are available equitably.

### Potential strengths and limitations

A key strength of the HAPPY study is that it is conducted in a diverse, multiethnic urban conurbation which will identify whether physical activity promotion and equitability differ across Greater London boroughs with different levels of deprivation. Findings will therefore potentially be representative of other diverse urban conurbations. A further strength is accelerometer assessed physical activity which will provide more accurate estimates of daily physical activity minimising the recall bias of participant self-report methods that typically lead to overestimates of physical activity.^37^ Furthermore, accelerometers worn on the non-dominant wrist and processing the raw accelerometer data in 5 s epochs will reduce effects of short intermittent outbursts (eg, random arm movements) included in the average acceleration values when calculating time spent in different PA intensities. This provides a more accurate measure of total PA. Potential limitations of the study include the cross-sectional design of the study; we will only be able to assess associations rather than cause and/or effect. Additionally, schools may not be representative of all schools in Greater London.

Global policies are focused on reducing physical inactivity by 15% by 2030,^2^ where inequalities may be best addressed during childhood when healthy behaviours can be embedded. The results of our study will therefore be of interest to public health policymakers, teachers, parents and children. The findings from the HAPPY study will determine how physically active school environments are associated with children’s physical activity, mental health and educational performance. This study has the potential to inform national strategies for the promotion of physical activity in schools, targeting all children and contributing towards reducing physical activity inequalities in child health.

## Supplementary material

10.1136/bmjopen-2025-103463 online supplemental file 1

10.1136/bmjopen-2025-103463 online supplemental file 2

10.1136/bmjopen-2025-103463 online supplemental file 3

## References

[1] WHO (2010). Global recommendations on physical activity for health.

[2] WHO (2018). Global action plan on physical activity 2018–2030: more active people for a healthier world.

[3] WHO (2021). Promoting physical activity through schools: a toolkit.

[4] WHO (2021). Making every school a health-promoting school: global standards and indicators.

[5] Marmot M (2017). Closing the health gap. Scand J Public Health.

[6] Marmot M, Allen J, Boyce T (2020). Health equity in England: the marmot review 10 years on.

[7] Hillier-Brown FC, Bambra CL, Cairns J-M (2014). A systematic review of the effectiveness of individual, community and societal level interventions at reducing socioeconomic inequalities in obesity amongst children. BMC Public Health.

[8] WHO (2016). Commission on Ending Childhood Obesity. Report of the commission on ending childhood obesity.

[9] UNESCO (2015). Quality physical education: policy guidelines: methodology.

[10] UNESCO (2015). Quality Physical Education (QPE): guidelines for policy makers.

[11] Department of Health and Social Care (2019). Physical activity guidelines: UK chief medical officers’ report.

[12] Sport England (2023). Children’s activity levels hold firm but significant challenges remain: active lives survey for children and young people.

[13] WHO (2021). Promoting physical activity through schools: policy brief.

[14] Ram B, Chalkley A, van Sluijs E (2021). Impact of The Daily Mile on children’s physical and mental health, and educational attainment in primary schools: iMprOVE cohort study protocol. BMJ Open.

[15] Venkatraman T, Honeyford K, Costelloe CE (2021). Sociodemographic profiles, educational attainment and physical activity associated with The Daily Mile registration in primary schools in England: a national cross-sectional linkage study. J Epidemiol Community Health.

[16] GOV UK (2024). Get information about schools.

[17] GOV UK (2014). National Curriculum.

[18] van Sluijs EMF, Skidmore PML, Mwanza K (2008). Physical activity and dietary behaviour in a population-based sample of British 10-year old children: the SPEEDY study (Sport, Physical activity and Eating behaviour: environmental Determinants in Young people). BMC Public Health.

[19] Adab P, Barrett T, Bhopal R (2018). The West Midlands ActiVe lifestyle and healthy Eating in School children (WAVES) study: a cluster randomised controlled trial testing the clinical effectiveness and cost-effectiveness of a multifaceted obesity prevention intervention programme targeted at children aged 6-7 years. Health Technol Assess.

[20] Corder K, Schiff A, Kesten JM (2015). Development of a universal approach to increase physical activity among adolescents: the GoActive intervention. BMJ Open.

[21] Corder K, Sharp SJ, Jong ST (2020). Effectiveness and cost-effectiveness of the GoActive intervention to increase physical activity among UK adolescents: A cluster randomised controlled trial. PLoS Med.

[22] Ram B, Foley KA, van Sluijs E (2022). Developing a core outcome set for physical activity interventions in primary schools: a modified-Delphi study. BMJ Open.

[23] Phillips LRS, Parfitt G, Rowlands AV (2013). Calibration of the GENEA accelerometer for assessment of physical activity intensity in children. J Sci Med Sport.

[24] Duncan MJ, Wilson S, Tallis J (2016). Validation of the Phillips et al. GENEActiv accelerometer wrist cut-points in children aged 5-8 years old. Eur J Pediatr.

[25] Scott JJ, Rowlands AV, Cliff DP (2017). Comparability and feasibility of wrist- and hip-worn accelerometers in free-living adolescents. J Sci Med Sport.

[26] Migueles JH, Rowlands AV, Huber F (2019). GGIR: A Research Community–Driven Open Source R Package for Generating Physical Activity and Sleep Outcomes From Multi-Day Raw Accelerometer Data. J Meas Phys Behav.

[27] Goodman R (1997). The Strengths and Difficulties Questionnaire: a research note. J Child Psychol Psychiatry.

[28] Muris P, Meesters C, van den Berg F (2003). The Strengths and Difficulties Questionnaire (SDQ)--further evidence for its reliability and validity in a community sample of Dutch children and adolescents. Eur Child Adolesc Psychiatry.

[29] CORC (2002). Strengths and Difficulties Questionnaire (SDQ).

[30] Breheny K, Passmore S, Adab P (2020). Effectiveness and cost-effectiveness of The Daily Mile on childhood weight outcomes and wellbeing: a cluster randomised controlled trial. *Int J Obes*.

[31] NFER Understanding age-related expectations UK: The National Foundation for Educational Research in England and Wales; 2002-2018.

[32] Robert C, Casella G (2004). Monte carlo statistical methods.

[33] Elm E von, Altman DG, Egger M (2007). Strengthening the reporting of observational studies in epidemiology (STROBE) statement: guidelines for reporting observational studies. *BMJ*.

[34] Monsalves MJ, Bangdiwala AS, Thabane A (2020). LEVEL (Logical Explanations & Visualizations of Estimates in Linear mixed models): recommendations for reporting multilevel data and analyses. BMC Med Res Methodol.

[35] WHO (2017). Health promoting schools.

[36] Public Health England (2020). What works in schools and colleges to increase physical activity? UK.

[37] DAPA measurement toolkit.

