# Supplementary material for: Exploring associations between active school environments and children’s physical activity, mental health and educational performance in Greater London primary schools: the Health and Activity of Pupils in the Primary Years (HAPPY) study protocol
Source: BMJ Open. 2025 Jul 28;15(7):e103463. doi: 10.1136/bmjopen-2025-103463 (PMC12306349; doi:10.1136/bmjopen-2025-103463)
Supplement: online supplemental file 1 [file bmjopen-15-7-s001.pdf]

Supplemental Table 2. Six domains of creating active school environments recommended by the World Health Organisation (WHO)

Domain	Description
1. Providing PA through quality physical education	Planned, progressive and inclusive learning experiences form the basis of quality physical education (QPE) that should be part of the curriculum throughout all the school years. QPE provides children with opportunities to be physically active during the school day, and has the potential to reach most children ensuring all have access to health promoting practices and embed healthier lifestyles.
2. Implementing strategies to encourage active travel to and from school	Active travel refers to making daily journeys through walking, cycling or other active means of transport. This includes public transport as these require physical activity (i.e., walking, cycling) to access these modes of transport. However, active travel excludes other motorised transport, e.g., cars, motorbikes, mopeds, etc. which require little or no physical activity to access.
3. PA opportunities before and after school	Physical activities that are provided before or after school are organised opportunities that take place outside of the school day (i.e., out-of-school-hours). These activities can be delivered by school staff, volunteers, or communities by externally funded non-profit or commercial organisations. These activities should be provided to all pupils at little to no-cost which would not exclude some children from benefitting.
4. Opportunities for PA at recess (i.e., breaks) and lunch	All pupils should have access to physical activity opportunities during scheduled school break and lunch periods. This could include the provision of built-in playground equipment, e.g., climbing frames, slides, overhead ladders (monkey bars), etc., and equipment that can be provided, e.g., footballs, tennis racquets, hoops, etc.
5. Active classrooms	Active classrooms reduce time spent being sedentary during lessons and can be incorporated during several lessons. These can include ‘movement breaks’ which is a short burst (3-5 minutes) of physical activity of different intensities during an otherwise sedentary lesson, e.g., jogging on the spot whilst solving a sum, counting steps whilst walking around the classroom.
6. PA for those with additional needs	PA provision in schools should be adapted to include all children in different stages of development, age, fitness, body size, and maturity. The activities should incorporate children’s abilities, needs, and capacity.
