# Supplementary material for: Exploring associations between active school environments and children’s physical activity, mental health and educational performance in Greater London primary schools: the Health and Activity of Pupils in the Primary Years (HAPPY) study protocol
Source: BMJ Open. 2025 Jul 28;15(7):e103463. doi: 10.1136/bmjopen-2025-103463 (PMC12306349; doi:10.1136/bmjopen-2025-103463)
Supplement: online supplemental file 3 [file bmjopen-15-7-s003.pdf]

Supplemental File 4. Parent, child and teacher questionnaire content

- Child questionnaire

Outcome	Measured by
Physical activity	- Active Lives Survey[1]<br>- Physical Activity Questionnaire for Children (PAQ-C)[2]<br>- SPEEDY[3]<br>- GoActive[4]
General health and wellbeing	- Child Health Utility 9D (CHU-9D)[5]<br>- EuroQol Visual Analogue Scale (EuroQol VAS)[6]<br>- UK Census 2021[7]
Demographic	- UK Census 2021[7] (age, sex, ethnic group)

- Parent questionnaire

Outcome	Measured by
Demographic	- UK Census 2021[7] (age, sex, ethnic group)
Socio-economic status	- UK Census 2021[7] (education, employment)
General health and wellbeing	- UK Census 2021[7]<br>- EuroQol Visual Analogue Scale (EuroQol VAS)[6]<br>- National Wellbeing[8]
Child mental health	- Strengths and Difficulties Questionnaire (SDQ)[9]
Child health	- UK Census 2021[7]
Household and accommodation	- UK Census 2021[7]

- Teacher questionnaire

Outcome	Measured by
Educational performance	• Age related expectations for reading, writing, math<br>• Focus and concentration

[1]Sport England: Active Lives Survey for Children and Young People https://www.sportengland.org/research-and-data/data/active-lives?section=measures#children-and-young-people-survey-9263
[2]Kowalski k, Crocker R and Donen R. The Physical Activity Questionnaire for Older Children (PAQ-C) and Adolescents (PAQ-Q) Manual. University of Saskatchewan
[3]van Sluijs EM, et al. Physical activity and dietary behaviour in a population-based sample of British 10-year old children: the SPEEDY study (Sport, Physical activity and Eating behaviour: Environmental Determinants in Young people). BMC Public Health. 2008
[4]Corder K, et al. Effectiveness and cost-effectiveness of the GoActive intervention to increase physical activity among UK adolescents: A cluster randomised controlled trial. PLoS Med. 2020
[5]Stevens KJ. Working with Children to Develop Dimensions for a Preference-Based, Generic, Paediatric, Health-Related Quality-of-Life Measure. *Qual Health Res.* 2010;20(3):340-351. DOI: 10.1177/1049732309358328
[6]EuroQol Research Foundation. EQ-5D-5L User Guide 2019
[7]UK Census United Kingdom. Office for National Statistics (ONS), 2021
[8]Measuring National Wellbeing – Personal Wellbeing. Office for National Statistics, 2019
[9]Goodman R. The Strengths and Difficulties Questionnaire: a research note. *Journal of Child Psychology and Psychiatry.* 1997;38(5):581-586. DOI: 10.1111/j.1469-7610.1997.tb01545.x
