# Supplementary material for: Exploring associations between active school environments and children’s physical activity, mental health and educational performance in Greater London primary schools: the Health and Activity of Pupils in the Primary Years (HAPPY) study protocol
Source: BMJ Open. 2025 Jul 28;15(7):e103463. doi: 10.1136/bmjopen-2025-103463 (PMC12306349; doi:10.1136/bmjopen-2025-103463)
Supplement: Supplementary file 4 [file bmjopen-2025-103463.reviewer_comments.pdf]

PEER REVIEW HISTORY

BMJ Open publishes all reviews undertaken for accepted manuscripts. Reviewers are asked to complete a checklist review form and are provided with free text boxes to elaborate on their assessment. These free text comments are reproduced below.

ARTICLE DETAILS

Title (Provisional)

Exploring associations between active school environments and children's physical activity, mental health, and educational performance in Greater London primary schools: The Health and Activity of Pupils in the Primary Years (HAPPY) study protocol

Authors

Ram, Bina; Gullett, Nancy; Benkhelfa, Amina; Cunningham, Mark; Taghavi Azar Sharabiani, Mansour; van Sluijs, Esther; Siddiqui, Nadia; Hillsdon, Melvyn; Summerbell, Carolyn; Pallan, Miranda; Saxena, Sonia

---

VERSION 1 - REVIEW

**Reviewer**	**1**
**Name**	**Roqué, Marta**
**Affiliation**	**Iberoamerican Cochrane Centre**
**Date**	**24-Apr-2025**
**COI**	**None**

Overall, the protocol is rigorous and well-written. A few comments:

1- Some typos (e.g. L187, L199) and use of both future and past tenses in the text.

2- I'd suggest to the authors to follow the LEVEL reporting guidelines to report the analyses and results of the multilevel models in the final paper. The methods are defined, but a bit more detail would be welcome (see level guidance). In one instance, clustering by classroom and school is mentioned (L 75), although in all the other instances only school clustering is considered (e.g. L 296) - please revise and correct.

3- Why an adult consent form? (L165)

4- Wrist-worn accelerometers were often considered unreliable tools to measure total PA, as they counted arm movements as total-body PA. Please, describe how the GENEActiv overcomes these reported limitations.

5- The power and sample size section is confusing. Figure 3 is not clearly described. The authors argue that Figure 3 shows the study to have enough power, but this conclusion is

based on the assumption that a >=0.55 effect size (minute increase in PA per unit increase in the AES) will be observed - how do you justify this assumption?

6- If I understand correctly, the sample size needed for an adequate power is 600 pupils, but the cross-sectional study aims to recruit 1000 pupils. What's the reasoning for that?

7- the authors don't delve in the potential confounding factors and ecologial bias potential in the analyses, beyond considering pupil's sex and ethnicity. They might mention potential confounding due to self-selection of participants in a specific school/class with higher socio-economic level, first language being English, already high PA levels, etc.

Reviewer **2**

Name **Brandes, Mirko**

Affiliation **Leibniz Institute for Prevention Research and Epidemiology, Prevention and Evaluation**

Date **07-Jun-2025**

COI **None**

Thank you for giving me the opportunity to review the study protocol. Overall, it is written very good, informative, and we definitively need more studies like this...

As it is a study protocol, there only a very few things to be addressed at this time point. The major thing: as required by BMJ, protocols should report on ongoing studies. With respect to the timeline provided in the paper, data collection has already been done (until Dec 2024). I will leave the decision, if this is still an "ongoing" study, to the editor (some other journal reject protocols if data collection is finished).

Some minor points:

- Methods, study design: if would suggest to include the overall timeline (Oct 2023 - Dec 2024) here, also details are provided later

- Outcomes and measures, line 254: please justify why you sampled at 85Hz

- Figure 1: please revise, you use different timelines on the left and on the right side of the figure, I suggest to use the same timeline for both study sections.

Finally, are more general point which can not be addressed since data collection has already finished, but maybe for consideration for the next study: after many years doing research in physical activity in pupils, I question (also myself) if accelerometry output, such as MVPA, is a really useful primary outcome. What do you think, would it also be worth to think about physical literacy, and put this as the primary outcome instead an artefactual measurement of one-week PA, which is obviously biased by the awareness of the children of the accelerometer on their wrist?

However, I am very interested in the results of the study!

VERSION 1 - AUTHOR RESPONSE

**Reviewer 1**			
1.	Some typos (e.g. L187, L199) and use of both future and past tenses in the text.	These have now been corrected on lines 187 and 199, and throughout the manuscript. References to data collection are past tense, and references to the analysis yet to be done is in future tense.	
2.	I'd suggest to the authors to follow the LEVEL reporting guidelines to report the analyses and results of the multilevel models in the final paper. The methods are defined, but a bit more detail would be welcome (see level guidance). In one instance, clustering by classroom and school is mentioned (L 75), although in all the other instances only school clustering is considered (e.g. L 296) - please revise and correct.	We thank the reviewer for their suggestion of using the LEVEL reporting guidelines in the final paper. We have now added this to the text.<br><br>We have revised our protocol throughout to clarify that our regression models will include clustering at school level as a random effect. We have added that we will conduct sensitivity analysis to include clustering at the class-level as an additional random effect.	<u>Page 15, lines 360-362</u><br>*“We will report our findings in line with the STROBE and LEVEL guidelines for reporting cross-sectional studies.”*<br><br><u>Page 15, lines 355-356</u><br>*“Sensitivity analysis will include clustering at class level within each school.”*
3.	Why an adult consent form? (L165)	The adult consent form was required as the adults (i.e., parents) were invited to complete a questionnaire which asked about their socio-demographic data, and to report their child’s mental health (see Supplemental File 3).	
4.	Wrist-worn accelerometers were often considered unreliable tools to measure total PA, as they counted arm movements as total-body PA. Please, describe how the GENEActiv overcomes these reported limitations.	We agree that random arm movements can inflate measures of total activity. To mitigate this, children were asked to wear the device on their non-dominant wrist (specified on page 9, line 208). In addition, the raw acceleration data was	<u>Methods: Page 11, lines 266-267</u><br>*“…raw data will be processed using 5-second epochs using the GGIR R-package.”*<br><br><u>Discussion: Pages 17, lines 395-398</u>

		processed in 5-seond epochs reducing the effect of short intermittent arm movements on average acceleration values, especially when calculating time spent in different intensities.<br><br>We have added further information to our methods and in the strengths and limitations section of the discussion.	*"Furthermore, accelerometers worn on the non-dominant wrist, and processing the raw accelerometer data in 5-second epochs will reduce effects of short intermittent outbursts (e.g., random arm movements) included in the average acceleration values when calculating time spent in different PA intensities. This provides a more accurate measure of total PA."*
5.	The power and sample size section is confusing. Figure 3 is not clearly described. The authors argue that Figure 3 shows the study to have enough power, but this conclusion is based on the assumption that a >=0.55 effect size (minute increase in PA per unit increase in the AES) will be observed - how do you justify this assumption?	Our effect size was based on a clinical target assuming a baseline of 60 baseline minutes of physical activity per pupil day recommended by the WHO guidelines for children aged <18 years. National guidance states that schools should contribute at least 30 of this 60 minutes to children's physical activity. Hence, we hypothesised that the schools' Active Environment Score (AES) would contribute to changes in this baseline, and that an effect size of approximately 0.50 would be clinically significant.<br><br>We also explored effect sizes between 0.3 and 1 that have previously been reported in studies of school-based PA interventions, and found that a total sample size of 600 pupils would be needed to detect an effect size of 0.55 with power of 80.<br><br>We have revised our sample size section to provide further clarity.	Pages 13/14, lines 320-323<br>*"We based our effect size of 60 minutes of PA per day (recommended global guidelines) of which 30 minutes should take place during the school day. We hypthosised that schools' Active Environment Score would contribute to changes in the baseline, thus, an effect size of 0.50 would be clinically significant. However, we also explored…"*
6.	If I understand correctly, the sample size needed for an adequate power is 600 pupils, but the cross-sectional study aims to	The sample size of 600 pupils was the minimum number required based on a conservative estimate of recruitment. However, we	Page 13, lines 314-316<br>*"The sample size of 600 pupils was the minimum number required for our power calculations; however we aimed*

	recruit 1000 pupils. What's the reasoning for that?	aimed to recruit more pupils to account for potential data losses (i.e., lost accelerometers, incomplete questionnaires, or poor data capture) that has occurred in previous studies.	*to recruit 1000 children to account for potential data losses (i.e., lost accelerometers, incomplete questionnaires, etc.).”*
7.	the authors don't delve in the potential confounding factors and ecologial bias potential in the analyses, beyond considering pupil's sex and ethnicity. They might mention potential confounding due to self-selection of participants in a specific school/class with higher socio-economic level, first language being English, already high PA levels, etc.	We will be conducting sensitivity analysis to include clustering by class (see Response #6). In addition, we had included a statement to include potential confounding for pre-specified school characteristics (e.g., first language not being English, and area-level deprivation, as well as key pupil-level characteristics including ethnicity, and sex) in our Statistical analysis section on page 14.	
**Reviewer 2**			
8.	As it is a study protocol, there only a very few things to be addressed at this time point. The major thing: as required by BMJ, protocols should report on ongoing studies. With respect to the timeline provided in the paper, data collection has already been done (until Dec 2024). I will leave the decision, if this is still an "ongoing" study, to the editor (some other journal reject protocols if data collection is finished).	Please see response to Q3 where we have now clarified that data collection is complete and that the data is being processed, cleaned, and coded in preparation for analysis. We will not start our analysis until the protocol has been accepted for publication.	
9.	Methods, study design: if would suggest to include the overall timeline (Oct 2023 - Dec 2024) here, also details are provided later	We have amended the text to include the overall timeline.	<u>Page 5, lines 125-126</u><br>*“…two components involving primary data collection between October 2023 and December 2024 and were conducted simultaneously (Figure 1).”*
10	- Outcomes and measures, line 254: please justify why you sampled at 85Hz	We have now added a sentence to explain why we sampled at 85Hz.	<u>Page 11, lines 264-265</u><br>*“…acceleration was sampled at 85.70Hz to allow for a measurement period of 9 days,*

			capturing a full week (7 days) of data."
11.	- Figure 1: please revise, you use different timelines on the left and on the right side of the figure, I suggest to use the same timeline for both study sections.	We have amended Figure 1 to use the same timeline for both study sections.	
12.	Finally, are more general point which can not be addressed since data collection has already finished, but maybe for consideration for the next study: after many years doing research in physical activity in pupils, I question (also myself) if accelerometry output, such as MVPA, is a really useful primary outcome. What do you think, would it also be worth to think about physical literacy, and put this as the primary outcome instead an artefactual measurement of one-week PA, which is obviously biased by the awareness of the children of the accelerometer on their wrist?	We thank the reviewer for sharing their thoughts on this interesting point.<br>We agree that physical literacy should be considered as a primary outcome in studies to overcome the bias in capturing a one-week objective measure of PA which is likely to be higher due to awareness of data on their PA being collected.	

VERSION 2 - REVIEW

Reviewer 2

Name Brandes, Mirko

Affiliation Leibniz Institute for Prevention Research and Epidemiology, Prevention and Evaluation

Date 28-Jun-2025

COI

I thank the authors for adequately addressing my comments, good luck with the HAPPY study.
